# Assessing spatial accessibility of rabies post-exposure prophylaxis clinics in Gansu Province, China

**DOI:** 10.3389/fpubh.2026.1826911

**Published:** 2026-06-03

**Authors:** Shuyu Liu, Na Jin, Xuefeng Liang, Jing An, Xiang Song, Xiaoshu Zhang

**Affiliations:** 1 Gansu Provincial Center for Disease Control and Prevention, Lanzhou, Gansu Province, China; 2 Northwest Institute of Eco-Environment and Resources, Chinese Academy of Sciences, Lanzhou, Gansu Province, China

**Keywords:** cost-distance analyses, Gansu Province, geographical accessibility, post-exposure prophylaxis (PEP) clinic, rabies

## Abstract

**Objective:**

To understand the current status of rabies post-exposure prophylaxis (PEP) clinic construction in Gansu Province and analyze the distribution of rabies PEP clinics across the province. To evaluate residents’ geographic accessibility to rabies PEP clinics, to provide a sound basis for the rational allocation of medical resources related to rabies PEP.

**Methods:**

Address information and service type data of rabies PEP clinics in Gansu Province in 2024 were collected. The number and kernel density of clinics were calculated by different areas and different services provided by clinics. Combined the road network, land cover, elevation, inland water body data downloaded from websites, and cost distance model in ArcGIS software was used to calculate the accumulated traffic-cost score for residents reaching the nearest rabies PEP clinic by different areas.

**Results:**

In 2024, Gansu Province had a total of 426 rabies PEP vaccination clinics. Among these, 69.72% provided wound management services, while 19.01% offered passive immunizing agents. Regional disparities were observed across different administrative and economic levels. In terms of spatial distribution, clinic density was highest in eastern regions, whereas western regions exhibited significantly lower density. Spatial analysis revealed that rabies PEP clinics in Gansu Province exhibited significant clustering characteristics, with a nearest neighbor ratio (NNR) of 0.490 (*Z* = −20.117, *p* < 0.05). Transportation accessibility assessment indicated that certain western and southern regions of Gansu Province had higher travel cost scores compared to other areas.

**Conclusion:**

Through analysis of the basic status of rabies exposure clinics in Gansu Province, combined with kernel density and transportation cost-distance analyses, reveals shortcomings in the standardized construction of rabies PEP clinics in the province. It is recommended to rationally plan clinic layouts, strengthen the service capacity building of clinics in economically underdeveloped and transportation-inconvenient areas, and enhance overall service capabilities.

## 1 Introduction

Rabies is an acute infectious disorder resulting from infection with the rabies virus. It predominantly impacts the central nervous system and is categorized as a zoonotic ailment, being globally widespread (1). Approximately 99% of human rabies cases are transmitted through dog bites (2). According to the 2018 statistical report by the World Health Organization (WHO), approximately 60,000 rabies-related deaths occur globally each year, with Asia and Africa accounting for 95% of these cases (3). In China, rabies is classified as a Category B notifiable infectious disease, and it continues to represent one of the nation’s most substantial public health challenges (3). It is noteworthy that reported cases of rabies consistently rank among the highest of all notifiable infectious diseases (3). During the period from 2007 to 2023, a cumulative total of 18,751 rabies cases were officially reported nationwide, corresponding to an average annual incidence rate of 0.08 per 100,000 population. The reported incidence rate has exhibited an overall declining trajectory; with 2011, 2018, and 2021 serving as pivotal dividing years, it has demonstrated a trend characterized by three consecutive phases of accelerated decline, followed by a subsequent deceleration in the downward pace (4).

Rabies is a lethal disease for which there is currently no efficacious cure. Indeed, once symptoms manifest, the mortality rate is invariably 100% (5). The distinguishing characteristic of this ailment is its preventable nature, in contrast to its current incurable status (6). Consequently, the most effective measure for preventing rabies is the prompt and standardized management of rabies exposure following injuries from cats, dogs, and other animals (7). As a vaccine-preventable disease, the prevention of rabies involves both pre- and post-exposure vaccination. It is imperative that high-risk groups, such as veterinarians and laboratory workers, receive post-exposure prophylaxis (PEP) vaccinations. In contrast, the majority of individuals require prompt post-exposure treatment, which is contingent on their exposure risk following potential rabies contact (5).

The WHO states that achieving an immunization coverage rate of 70% among dogs is essential for effectively controlling and eliminating rabies (8–10). However, vaccination of dogs and cats is neither mandatory nor widely implemented in China, resulting in significantly lower immunization rates than the WHO-recommended threshold of 70% (11). Consequently, rabies prevention and control in China currently relies primarily on public awareness and post-exposure prophylaxis (PEP). In December 2015, the World Health Organization (WHO) and its partners established a framework to eliminate human rabies by 2030. Under this framework, Chinese experts have called for the elimination of human rabies in China by 2020. As a country with a high burden of rabies, China must implement multiple measures to achieve this, including enhanced management of cats and dogs, improved post-exposure prophylaxis protocols and increased PEP accessibility (12). Research indicates that the distance between individuals who have been exposed to rabies and clinics providing PEP affects their ability to receive timely vaccination for prevention (13). Therefore, understanding the distribution and geographic accessibility of these clinics provides a theoretical basis for the rational allocation of related healthcare resources and for improving their accessibility.

The geographical accessibility of healthcare resources denotes the degree of convenience for the population to access specific healthcare services. It is typically measured by travel distance, travel time, or the availability of allocated resources within a particular area (13, 14). The spatial distribution of vaccination clinics is of crucial significance in ensuring equitable access to immunization services. Geographic Information System (GIS) technology serves as a potent instrument for visualizing the spatial distribution patterns of vaccination resources and discerning disparities in resource allocation. This approach provides an evidence-based framework for optimizing resource distribution. The use of GIS to investigate the spatial allocation of vaccination resources has emerged as a research focus within the academic community (15–20).

As a northwestern inland province with complex terrain (plateaus, mountains, and rural hinterlands), Gansu has a large population of free-roaming dogs and low rabies vaccination coverage in rural areas, lacking an effective immune barrier that facilitates rabies virus circulation among local animals. Rabies remains one of the most fatal infectious diseases in Gansu, with a 100% case fatality rate; Data from the Chinese Center for Disease Control and Prevention (China CDC) show a total of 44 cases were reported from 2013 to 2024 across 8 prefectures with specific annual distributions, and all human rabies cases have been traced back to dogs (including both domestically owned unvaccinated dogs and stray dogs), which aligns with the overall epidemiological trend in China where approximately 95% of rabies cases are caused by dogs posing a severe threat to public health and highlighting the critical need for targeted research and prevention strategies. So Gansu Province is selected as the rabies research area for its unique epidemiological transition, geographic vulnerability, and public health urgency.

In this study, baseline data of PEP clinics across the province were collected. PEP clinics across different regions were classified into four types: (1) provision of rabies vaccine alone; (2) provision of rabies vaccine combined with wound treatment; (3) provision of rabies vaccine combined with passive immunization preparations; (4) provision of rabies vaccine combined with both wound treatment and passive immunization preparations. Among them, in rabies-prone areas such as rural and mountainous regions, basic clinics providing vaccination are given priority, because these areas have low population density and inconvenient transportation, it is necessary to prioritize ensuring that exposed individuals can quickly obtain vaccine immunization to reduce the risk of virus invasion. In contrast, comprehensive clinics are mainly distributed in areas with concentrated medical resources such as county towns and urban areas, meeting the subsequent vaccination needs of exposed individuals, forming a clinic layout of “basic emergency response and full-course prevention and control” that adapts to the geographical distribution and incidence characteristics of Gansu.

To characterize the spatial distribution patterns of these PEP clinics, GIS-based analytical methods, including average nearest neighbor analysis, kernel density estimation, and cost distance modeling, were utilized to characterize the spatial distribution patterns. Additionally, the study assesses the geographical accessibility of residents to rabies PEP services, aiming to provide a theoretical foundation for the rational allocation of rabies PEP related medical resources in Gansu Province.

## 2 Materials and methods

### 2.1 Description of the study area

The study area, Gansu Province, is situated in northwestern China, at the junction of the Loess Plateau, the Inner Mongolian Plateau, and the Qinghai-Tibet Plateau. It features diverse landforms, including mountainous areas, plateaus, plains, river valleys, deserts, and Gobi regions, with a correspondingly diverse climate encompassing multiple climate types. Gansu Province covers a total area of 42.59 × 10^4^ km^2^. As of the end of 2023, its permanent resident population stood at 24.65 million. Administratively, the province governs 14 prefecture-level cities (prefectures) and Lanzhou New Area, consisting of 86 county-level administrative divisions in total, including counties, county-level cities, and urban districts.

Evidently, the economic development of Gansu Province is constrained by inherent natural conditions, leading to relatively underdeveloped and unevenly distributed regional economic activities. The eastern Loess Plateau area is characterized by a relatively high population density, yet it faces significant transportation challenges due to complex topographical conditions. In contrast, the western region, which belongs to the arid desert zone of northwestern China, is sparsely populated and economically underdeveloped.

**Figure 1:**
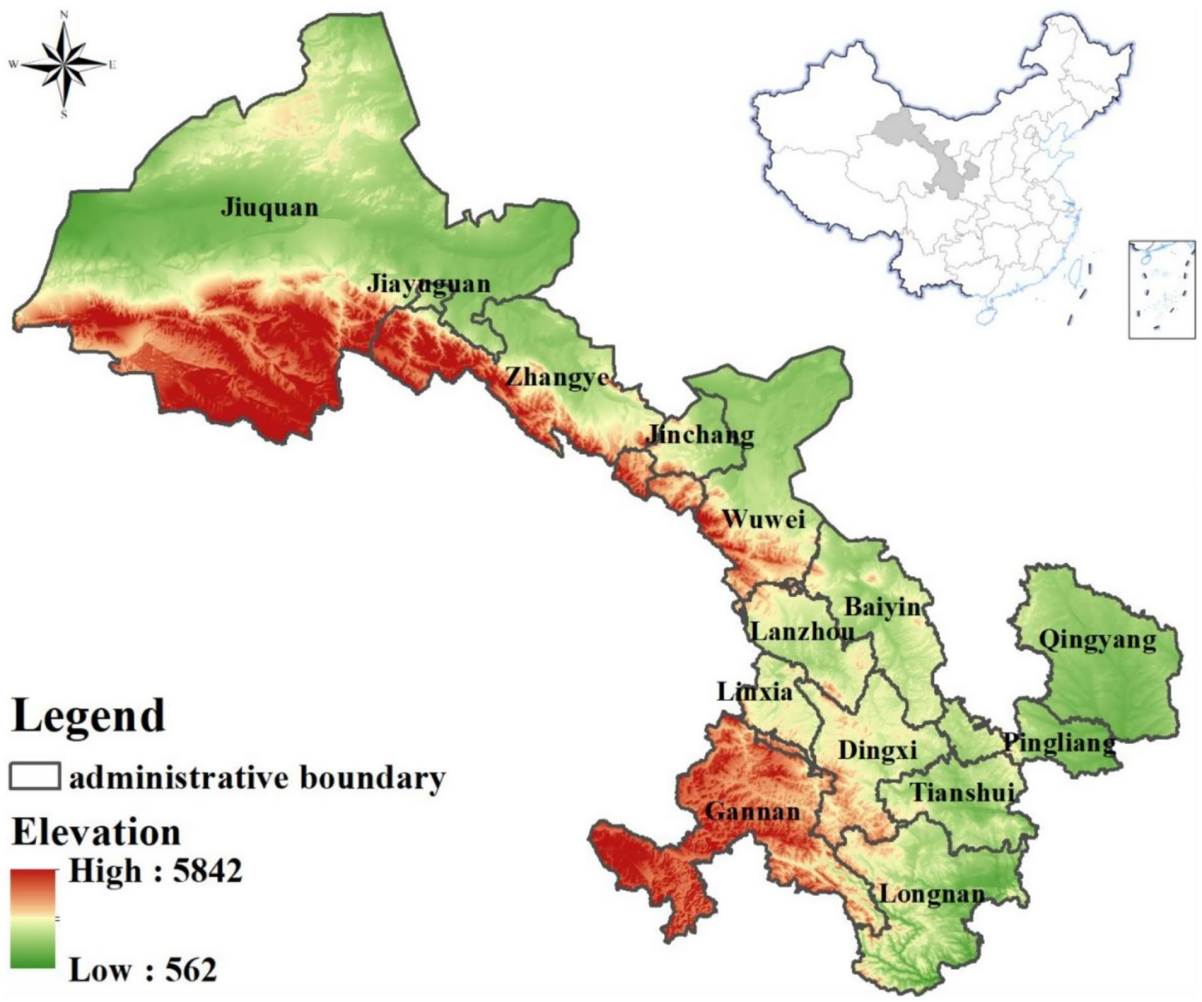
Overview of the study area in Gansu Province.

Human rabies cases have been reported in Gansu Province in most years. Data from the Chinese Center for Disease Control and Prevention (China CDC) show that during the period from 2007 to 2023, the annual number of cases remained at a low level, presenting an overall low-level fluctuating trend (5). To date, Gansu Province has established 2,559 vaccination service units, among which 426 are dedicated post-exposure prophylaxis (PEP) clinics for rabies (Figure 1).

### 2.2 Data collection and pre-processing

The datasets employed in this study comprise population data, geographic information data, remote sensing data, and PEP data. Population data encompass two core components: population size and population density. Population size data were derived from the Basic Information Subsystem of the Information Management System at the Chinese Center for Disease Control and Prevention (China CDC). Population density data, in contrast, were retrieved from the 2020 WorldPop dataset,^1^ specifically 1 km-resolution GeoTIFF raster files. This population distribution layer was constructed via a random forest model developed by Andrea et al., which integrates statistical census data, digital elevation models (DEMs), nighttime light imagery, and hydrological network data (21–23). Geographic information data includes 1:250,000-scale county-level administrative boundaries for Gansu Province provided by the Chinese Center for Disease Control and Prevention, settlement data sourced from the National Geographic Information Resource Catalog Service System (24), and road network vector data obtained from OpenStreetMap (OSM), an open-access mapping platform (25). Remote sensing data includes land cover data obtained from the European Space Agency (ESA) official website^2^ in GeoTIFF format with a spatial resolution of 300 meters, and a digital elevation model (DEM) derived from the Shuttle Radar Topography Mission (SRTM) conducted by the National Aeronautics and Space Administration (NASA) with a spatial resolution of 3 arc-seconds. For subsequent analysis, the datasets were clipped against the administrative boundaries of Gansu Province to derive provincial-scale data, which were subsequently reprojected to the CGCS2000-based ALBERS equal-area conic projection. The adopted projection parameters are as follows: central meridian (to be specified); latitude of origin: 0°N; standard parallel 1: 25°N; standard parallel 2:47°N. As previously noted, all raster datasets were uniformly resampled to a spatial resolution of 500 meters. Subsequently, DEM data were used to derive slope datasets.

Rabies PEP clinic data for Gansu Province were retrieved from the Gansu Provincial Immunization Program Information System and the Standard Code Management Module of the China CDC, encompassing key variables including institution names, addresses, vaccine inoculation volumes, staff counts, and the number of inoculation stations, among others. Geocoding of spatial coordinates for each PEP clinic was performed in ArcGIS based on their respective addresses, with spatial mapping conducted within the administrative boundaries of Gansu Province. Additionally, 2024 annual rabies vaccination dose records for each clinic were extracted from the Gansu Provincial Immunization Program Management System for subsequent analysis.

This study has been reviewed and approved by the Gansu Provincial Center for Disease Control and Prevention Medical Ethics Committee (Gansu CDC lun No. [026] of 2023).

### 2.3 Research methods

#### 2.3.1 Basic characteristics and spatial distribution of rabies PEP clinics

The present study conducted an analysis of 426 rabies PEP clinics in Gansu Province during the year 2024. For each administrative region, three key metrics were calculated: The first variable is the per capita clinic availability, which is measured as the number of clinics per 100,000 population. The second variable is the clinic density, which is measured as the number of clinics per 10,000 km^2^. The third variable is the vaccination staff allocation, both per capita and per unit area. The metrics in question were computed by dividing the number of clinics and staff by the corresponding regional population and administrative area data.

Gansu Province was divided into three regions based on geographical location: the eastern region (comprising Tianshui, Pingliang, Qingyang, and Longnan), the central region (comprising Baiyin, Lanzhou, Dingxi, Linxia, and Gannan), and the western region (comprising Jiuquan, Jiayuguan, Jinchang, Zhangye, and Wuwei). The categorization of the province was based on regional GDP, with the result that the province was divided into three income tiers. The first tier comprised high-income regions, which included Lanzhou, Qingyang, Jiuquan, Tianshui, and Wuwei. The second tier comprised middle-income regions, which included Baiyin, Pingliang, Zhangye, Longnan, and Dingxi. The third and final tier comprised low-income regions, which included Jinchang, Jiayuguan, Linxia, and Gannan. The service capabilities of vaccination clinics across different regions were classified into four types: The provision of the rabies vaccine can be categorized into four distinct approaches: firstly, the provision of the rabies vaccine in isolation; secondly, the provision of the rabies vaccine in combination with wound treatment; thirdly, the provision of the rabies vaccine in combination with passive immunization preparations; and fourthly, the provision of the rabies vaccine in combination with both wound treatment and passive immunization preparations. Chi-square tests were utilized to assess differences in service type composition across different groups. The tests were conducted under the null hypothesis that no difference existed in service type composition among the groups, with a significance level set atα = 0.05. The Chi-square test statistic (*χ*^2^) and corresponding *p*-values were calculated to evaluate the statistical significance of observed differences.

#### 2.3.2 Spatial accessibility analysis

The Average Nearest Neighbor (ANN) analysis assesses the spatial distribution pattern of vaccination clinics by comparing the observed mean nearest neighbor distance (Do) with the expected mean nearest neighbor distance (De) under a random distribution (26). The ANN index is computed as:

ANN=Do/De

Interpretation of ANN results: ANN < 1 indicates a clustered spatial distribution of vaccination clinics; ANN > 1 suggests a dispersed spatial distribution; ANN = 1 implies a random spatial distribution (27). This method facilitates the determination of whether vaccination clinics are concentrated in specific areas, evenly distributed, or randomly located across the study region.

Kernel Density Estimation (KDE) is a non-parametric spatial analysis method widely applied in epidemiological research for density-based inference. As an effective tool for characterizing spatial distribution patterns, KDE can quantify the density distribution of vaccination clinics while incorporating both geographical proximity and service capacity (28). The present study employed the spatial analysis module provided by ArcGIS software to compute kernel density, setting a 50 k search radius for the PEP kernel density analysis with a spatial resolution of 500 m. Subsequently, the calculated kernel density underwent a common logarithmic transformation, and spatial statistical tools were utilized to compute the geometric mean and geometric standard deviation of kernel density across different administrative regions.

#### 2.3.3 Transportation cost analysis

Transportation cost analysis was conducted using the Cost Distance Analysis module in ArcGIS software. Specifically, the transportation cost for residents at each cell location to access the nearest PEP clinic was quantified by computing the cumulative cost assigned to each cell (representing a resident location) in the cost raster from its nearest source cell (representing a PEP clinic location).

The cost layer was generated by integrating transportation network vector data and land cover data. First, transportation network vector data were converted into raster data by road type, with a spatial resolution of 500 m. Subsequently, a road speed layer was derived by reclassifying the raster data based on the travel speeds corresponding to different road types. Table 1 presents the maximum travel speeds for various road categories (29, 30). The maximum walking speeds for different land cover types were obtained from existing literature (Table 2) (31–34). Given that slope influences walking speed, Tobler’s hiking function was employed to calculate walking speeds across different slope gradients, with the formula as follows (35):

Walking speed=6×exp(−3.5×∣tan(Slope/57.296)+0.05∣)

**Table 1:** Road types in OSM road data, corresponding travel modes, and maximum trafficability speeds.

Road type	Travel mode	Speed
Motorway	Motor vehicle	120
Trunk	Motor vehicle	80
Secondary	Motor vehicle	80
Primary	Motor vehicle	80
Trunk_link	Motor vehicle	60
Secondary_link	Motor vehicle	60
Primary_link	Motor vehicle	60
Motorway_link	Motor vehicle	60
Tertiary	Motor vehicle	40
Unclassified	Motor vehicle	30
Tertiary_link	Motor vehicle	30
Turning circle	Motor vehicle	30
Residential	Mixed human-vehicle	20
Service	Mixed human-vehicle	20
Road	Mixed human-vehicle	20
Pedestrian	Non-motorized bicycle	10
Cycleway	Non-motorized bicycle	10
Footway	Pedestrian travel	5
Services	Pedestrian travel	5
Path	Pedestrian travel	5
Living street	Pedestrian travel	5
Rest area	Pedestrian travel	5
Track	Pedestrian travel	5
Bus stop	Pedestrian travel	5
Brideway	Pedestrian travel	5
Trail	Pedestrian travel	5
Steps	Pedestrian travel	5
Construction	Non-traversable	0
Raceway	Non-traversable	0
Proposed	Non-traversable	0
Bus guideway	Non-traversable	0
Escape	Non-traversable	0
Via_ferrate	Non-traversable	0

**Table 2:** European space agency (ESA) land cover data type codes, type descriptions, and corresponding maximum trafficability speeds.

Code	Land cover type description	Speed
11	Post-flooding or irrigated croplands (or aquatic)	5
14	Rainfed croplands	5
20	Mosaic cropland(50–70%)/vegetation(grassland/shrub land/forest)(20–50%)	5
30	Mosaic vegetation(grassland/shrub land/forest)(50–70%)/cropland(20–50%)	5
40	Closed to open (>15%) broad leaved evergreen or semi-deciduous forest (>5 m)	5
50	Closed (40%) broad leaved deciduous forest (>5 m)	5
60	Open (15–40%) broad leaved deciduous forest/woodland (>5 m)	5
70	Closed (40%) needle leaved evergreen forest (>5 m)	5
90	Open (15–40%) needle leaved deciduous or evergreen forest (>5 m)	5
100	Closed to open (>15%) mixed broad leaved and needle leaved forest (>5 m)	5
110	Mosaic forest or shrub land (50–70%) / grassland (20–50%)	3
120	Mosaic grassland (50–70%) / forest or shrub land (20–50%)	3
130	Closed to open (>15%)(broad leaved or needle leaved, evergreen or deciduous) shrub land (<5 m)	5
140	Closed to open(>15%) herbaceous vegetation (grassland, savannas or lichens/mosses)	3
150	Sparse(<15%)vegetation	4
170	Closed(>40%) broad leaved forest or shrub land permanently flooded-Saline or brackish water	0
180	Closed to open (>15%) grassland or woody vegetation on regularly flooded or waterlogged soil—Fresh, brackish or saline water	0
190	Artificial surfaces and associated areas (Urban areas >50%)	5
200	Bare areas	2
210	Water bodies	0
220	Permanent snow and ice	0

A Land Cover-Slope Speed layer was generated by multiplying the slope-adjusted walking speed by a corresponding normalization coefficient (X). This coefficient X was derived by dividing the maximum walking speed for each land cover type by 5.04 km/h (the maximum walking speed in flat areas, as calculated from Tobler’s formula).

The maximum value composition method was employed to integrate the road speed layer and the Land Cover-Slope Speed layer, yielding a comprehensive traffic speed layer. Further, a traffic cost score layer was generated by calculating the traffic cost score—representing the traversal difficulty of each grid cell—based on traffic speed. The calculation formula is as follows (36):

Traffic cost score=130/(traffic velocity+10)

Subsequently, cost-distance analysis was performed with rabies PEP clinic data designated as the source (i.e., destinations) and the traffic cost score layer as the cost raster. This analysis produced a layer representing the actual cumulative transportation cost scores for residents at each cell location to reach the nearest PEP clinic. These scores were then transformed using the common logarithm (i.e., base-10 logarithm). Finally, spatial statistics tools were utilized to compute the geometric mean and geometric standard deviation of the logarithmically transformed cumulative transportation cost scores for accessing the nearest PEP clinic across different administrative regions.

## 3 Results

### 3.1 Composition of rabies PEP outpatient types

**Figure 2:**
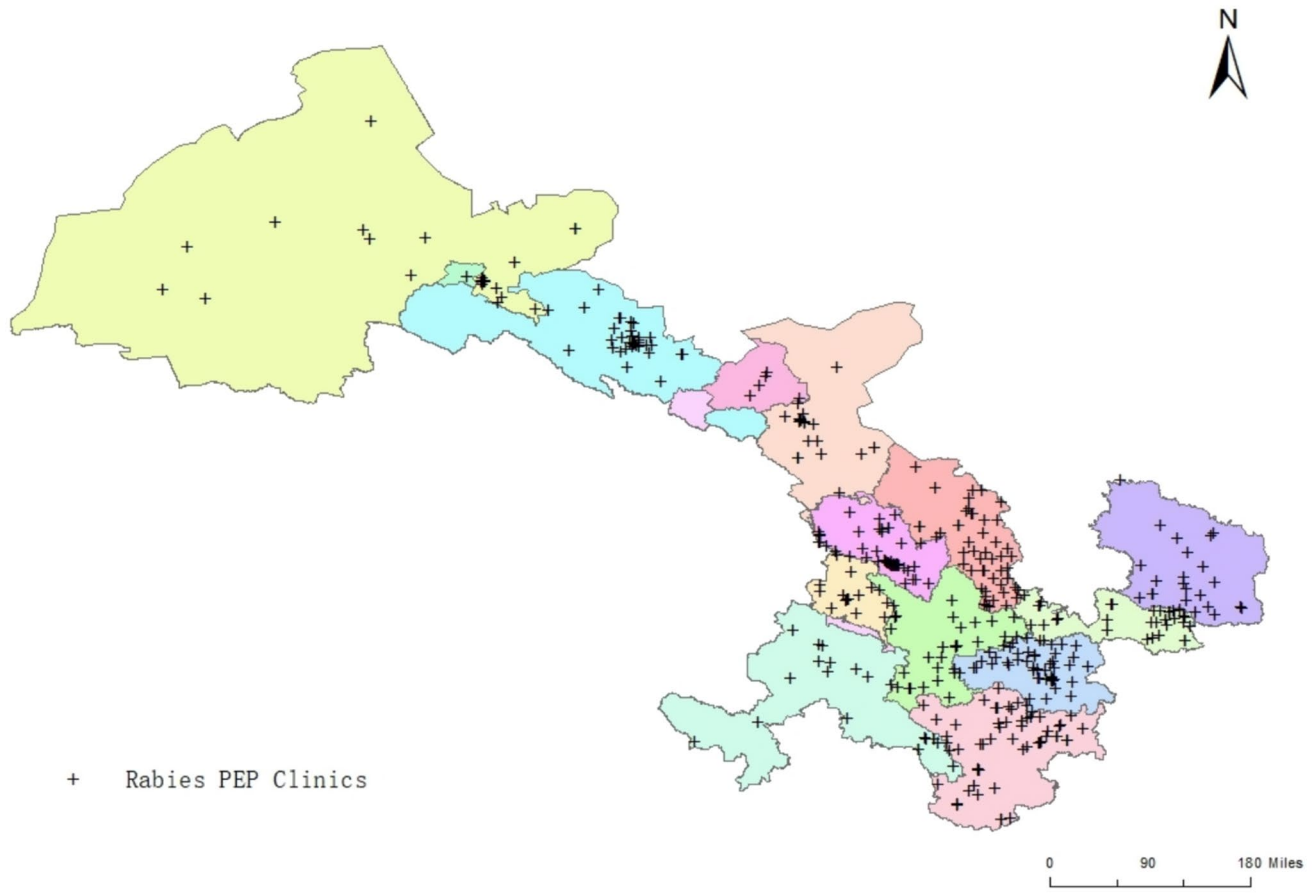
Spatial distribution of rabies PEP clinics in Gansu Province.

In this study, we collated basic information on 426 rabies PEP clinics across 14 prefecture-level cities in Gansu Province. Within these regions, Tianshui, Longnan, Lanzhou, Baiyin, and Dingxi each had over 40 rabies PEP clinics, while Jiayuguan and Jinchang had fewer than 10. Jiayuguan demonstrated the lowest number of clinics, with a single facility. In terms of the composition of the types of rabies PEP clinics, 29.58% (126 clinics) provide only vaccination services, 51.41% (219 clinics) offer basic “vaccine + wound treatment” services, while merely 18.08% (77 clinics) are capable of providing comprehensive “vaccine + wound treatment + passive immunizing agent” services. 69.72% of the rabies PEP clinics in Gansu province can provide wound treatment services, and 19.01% can provide passive immunization preparations. Among rabies PEP clinics in Gansu Province, 64.32% provide wound management services, whereas a mere 19.01% are equipped with passive immunotherapy agents— a proportion markedly lower than that observed in eastern Chinese provinces. Specifically, six prefecture-level cities in Gansu reported a wound management service provision rate of over 80% among local rabies PEP clinics, with Jiayuguan and Jinchang achieving full coverage of this service across all their clinics. In striking contrast, Tianshui, which hosts the largest number of rabies PEP clinics in the province, had the lowest service provision rate of only 28.33% for wound management, a figure that is significantly inferior to those of other prefecture-level cities in Gansu (Figure 2; Table 3).

**Table 3:** Number and composition of rabies PEP clinics in Gansu Province.

Prefecture	Total clinics (*n*)	Number and service type of rabies PEP clinics (*n*, %)				Clinics providing wound management (*n*, %)	Clinics providing passive immunization (*n*, %)	*χ* ^2^	p
		Only vaccine	Vaccine + Wound care	Vaccine + Passive immunization	Vaccine + Wound care + Passive immunization				
Baiyin	46	13 (28.26)	32 (69.57)	0 (0.00)	1 (2.17)	33 (71.74)	1 (2.17)	–	–
Dingxi	42	12 (28.57)	25 (59.52)	0 (0.00)	5 (11.90)	30 (71.43)	5 (11.90)		
Gannan	18	7 (38.89)	8 (44.44)	1 (5.56)	2 (11.11)	10 (55.56)	3 (16.67)		
Jiayuguan	1	0 (0.00)	0 (0.00)	0 (0.00)	1 (100.00)	1 (100.00)	1 (100.00)		
Jinchang	5	0 (0.00)	2 (40.00)	0 (0.00)	3 (60.00)	5 (100.00)	3 (60.00)		
Jiuquan	27	11 (40.74)	8 (29.63)	0 (0.00)	8 (29.63)	16 (59.26)	8 (29.63)		
Lanzhou	48	13 (27.08)	19 (39.58)	0 (0.00)	16 (33.33)	36 (75.00)	16 (33.33)		
Linxia	15	1 (6.67)	8 (53.33)	0 (0.00)	6 (40.00)	14 (93.33)	6 (40.00)		
Longnan	54	10 (18.52)	38 (70.37)	1 (1.85)	5 (9.26)	43 (79.63)	6 (11.11)		
Pingliang	28	2 (7.14)	17 (60.71)	0 (0.00)	9 (32.14)	26 (92.86)	9 (32.14)		
Qingyang	24	1 (4.17)	14 (58.33)	0 (0.00)	9 (37.50)	23 (95.83)	9 (37.50)		
Tianshui	60	41 (68.33)	12 (20.00)	2 (3.33)	5 (8.33)	17 (28.33)	7 (11.67)		
Wuwei	22	9 (40.91)	10 (45.45)	0 (0.00)	3 (13.64)	13 (59.09)	3 (13.64)		
Zhangye	36	6 (16.67)	26 (72.22)	0 (0.00)	4 (11.11)	30 (83.33)	4 (11.11)		
Administrative region									
Eastern Region	166	54 (32.53)	81 (48.80)	3 (1.81)	28 (16.87)	109 (65.66)	31 (18.67)	21.64	<0.05
Central Region	169	46 (27.22)	92 (54.44)	1 (0.59)	30 (17.75)	123 (72.78)	31 (18.34)		
Western Region	91	26 (28.57)	46 (50.55)	0 (0.00)	19 (20.88)	65 (71.43)	19 (20.88)		
Economic level									
High	181	75 (41.44)	63 (34.81)	2 (1.10)	41 (22.65)	105 (58.01)	43 (23.76)	31.73	<0.05
Middle	206	43 (20.87)	138 (66.99)	1 (0.49)	24 (11.65)	162 (78.64)	25 (12.14)		
Low	39	8 (20.51)	18 (46.15)	1 (2.56)	12 (30.77)	30 (76.92)	13 (33.33)		
Total	426	126 (29.58)	219 (51.41)	4 (0.94)	77 (18.08)	297 (69.72)	81 (19.01)		

Regionally stratified analysis identified pronounced disparities in the distribution and service capacity of rabies post-exposure prophylaxis (PEP) clinics across Gansu Province. Specifically, the eastern, central and western regions housed 166, 169 and 91 rabies PEP clinics, respectively, with the western region recording the lowest clinic count. For wound management service provision, the central region had the highest proportion of clinics offering this service (72.78%), followed by the western region (71.43%), while the eastern region had the lowest (65.66%). In terms of passive immunization agent provision, the western region had the highest proportion of clinics with such supplies (20.88%). A chi-square test verified statistically significant differences in the composition of service types across the three regional strata (*χ*^2^ = 21.64, *p* < 0.05) (Table 3).

Stratification by economic development level also revealed distinct patterns in the distribution of rabies PEP clinics and their service configurations. High-economic-level areas had a total of 181 rabies PEP clinics, among which those providing vaccination services only constituted the largest proportion (41.44%); additionally, the proportion of clinics equipped with passive immunization agents was relatively high in these areas, at 23.76%. In contrast, medium-economic-level areas contained 206 clinics, where 66.99% provided combined vaccination plus wound management services, which represented the dominant service model in this stratum. Low-economic-level regions had 39 rabies PEP clinics in total: 76.92% of these clinics offered wound management services, a comparatively high proportion, and crucially, the proportion of clinics providing passive immunization agents was the highest across all economic strata, reaching 33.33%. A chi-square test further demonstrated statistically significant differences in the composition of service types among areas with different economic development levels (*χ*^2^ = 31.73, *p* < 0.05) (Table 3).

### 3.2 Resource allocation and spatial distribution characteristics of rabies PEP clinics in Gansu Province

In 2024, a total of 426 rabies PEP clinics were in operation in Gansu Province, with 3,876 healthcare professionals assigned to these facilities. Regarding the spatial distribution of clinics, when measured by the number of clinics per 100,000 population, Zhangye had the highest allocation (3.18), whereas Jiayuguan had the lowest (0.32), representing a nearly 10-fold difference between the two regions. In terms of clinic density (per 10,000 km^2^), Tianshui exhibited the highest density (41.58), while Jiuquan had the lowest (1.42), which indicated a prominent unbalanced regional distribution pattern. From the perspective of functional classification, wound management-only clinics dominated in most regions, while the quantity of passive immunization-only clinics was generally insufficient. Specifically, Baiyin had the lowest density of passive immunization-only clinics (0.47/10,000 km^2^), while Jiayuguan had the highest (3.41/10,000 km^2^), suggesting significant disparities in post-exposure passive immunization service capabilities across different regions.

The allocation of vaccinators is a key factor affecting the accessibility of rabies PEP services. There were significant differences in the total quantity and spatial distribution of vaccinators among the 14 prefectures in Gansu Province. Gannan had the highest number of vaccinators per 10,000 population (4.35), while Linxia City had the lowest (0.43). In terms of vaccinator density per 10,000 km^2^, Lanzhou ranked the highest (256.82), whereas Jiuquan was the lowest (13.26).

From the perspective of administrative divisions, significant differences in rabies prevention and control resources and vaccination status were identified among the Eastern, Central, and Western regions of Gansu Province. In terms of clinic density, the Eastern Region had the highest value (20.61/10,000 km^2^), while the Western Region had the lowest (3.30/10,000 km^2^). Regarding vaccinator allocation, the Western Region had the highest number of vaccinators per 10,000 population (1.76), whereas the Central Region had the lowest (1.45). In terms of total rabies vaccine doses administered, the Eastern Region had the highest volume (147,774 doses) with an average daily dose of 404.86, while the Western Region had the lowest total doses (70,770 doses) with an average daily dose of 193.89. The Eastern Region also had the highest per capita daily dose (0.27), and the Central Region had the lowest (0.22). Combined with population density data, the Eastern Region had the highest population density (116.94/km^2^), while the Western Region had the lowest (15.95/km^2^). These findings indicate that the difference in regional population agglomeration is one of the key factors leading to disparities in the allocation of prevention and control resources and the volume of vaccine administration.

In terms of economic development levels, the characteristics of rabies prevention and control varied significantly among high, middle, and low economic level regions. Regarding clinic resources, the middle economic level regions had the highest number of clinics per 100,000 population (2.19) and clinic density (16.97/10,000 km^2^), while the low economic level regions had the lowest values (1.10 and 6.66, respectively). In terms of vaccinator allocation, the middle economic level regions had the highest number of vaccinators per 10,000 population (1.87), and the low economic level regions had the lowest (1.34). For vaccine administration indicators, the high economic level regions had significantly higher total vaccine doses administered (198,092 doses), average daily doses (542.72), and per capita daily doses (0.33) compared with the middle and low economic level regions, while the low economic level regions had the lowest performance in all vaccine-related indicators. This suggests that the level of economic development exerts a significant impact on the investment in rabies prevention and control resources and the demand for prevention and control services. Economically developed regions typically have stronger public awareness of rabies prevention, more sufficient prevention and control resources, and higher demand for vaccination.

Based on the spatial coordinates of rabies PEP clinics in Gansu Province and the clinic distribution data presented in Table 4, average nearest neighbor analysis was conducted to quantitatively characterize the spatial distribution pattern of the clinics. The results showed that the nearest neighbor ratio (NNR) was 0.490 (Z = −20.117, *p* < 0.05). Since the NNR value was less than 1, it indicated that the spatial distribution of rabies PEP clinics in Gansu Province exhibited obvious agglomeration characteristics. This agglomeration feature was consistent with the regional differences in clinic density reflected in Table 4: Tianshui and Lanzhou had the highest clinic densities (41.58/10^4^ km^2^ and 36.69/10^4^ km^2^, respectively), while Jiuquan and Gannan had the lowest densities (1.42/10^4^ km^2^ and 4.67/10^4^ km^2^, respectively), thereby demonstrating a significant uneven spatial distribution.

**Table 4:** Basic profile of rabies PEP clinic services in Gansu Province.

Prefecture	Clinics (10^5^ population)			Clinic density (10^4^ km^2^)			Vaccinators			Rabies vaccine doses administered			Population density (/km^2^)
	Total clinics	Wound management only clinics	Passive immunization only clinics	Total clinics	Wound management only clinics	Passive immunization only clinics	Total	Per 10^4^ population	per 10^4^ km^2^	Total	Average daily doses	Per capita daily doses	
Baiyin	3.04	2.18	0.07	21.69	15.56	0.47	429	2.84	202.26	16,509	45.23	0.11	71.29
Dingxi	1.66	1.19	0.20	20.66	14.76	2.46	463	1.83	227.74	21,981	60.22	0.13	124.16
Gannan	2.60	1.45	0.43	4.67	2.60	0.78	301	4.35	78.14	5,410	14.82	0.05	17.96
Jiayuguan	0.32	0.32	0.32	3.41	3.41	3.41	24	0.77	81.77	8,725	23.90	1.00	106.53
Jinchang	1.14	1.14	0.68	5.57	5.57	3.34	61	1.39	67.99	7,308	20.02	0.33	48.82
Jiuquan	2.56	1.52	0.76	1.42	0.84	0.42	252	2.39	13.26	22,390	61.34	0.24	5.56
Lanzhou	1.10	0.83	0.37	36.69	27.52	12.23	336	0.77	256.82	75,533	206.94	0.62	333.21
Linxia	0.71	0.66	0.28	18.36	17.14	7.34	91	0.43	111.40	12,462	34.14	0.38	258.26
Longnan	2.24	1.79	0.25	19.40	15.45	2.16	588	2.44	211.22	36,919	101.15	0.17	86.47
Pingliang	1.51	1.41	0.49	25.07	23.28	8.06	108	0.58	96.70	26,111	71.54	0.66	165.51
Qingyang	1.10	1.06	0.41	8.85	8.48	3.32	219	1.00	80.76	41,435	113.52	0.52	80.38
Tianshui	2.01	0.57	0.23	41.58	11.78	4.85	567	1.90	392.90	43,309	118.65	0.21	206.82
Wuwei	1.50	0.89	0.20	6.62	3.91	0.90	266	1.82	80.03	15,425	42.26	0.16	44.07
Zhangye	3.18	2.65	0.35	8.82	7.35	0.98	171	1.51	41.89	16,922	46.36	0.27	27.70
Administrative region													
Eastern Region	1.76	1.16	0.33	20.61	13.53	3.85	1,482	1.57	183.97	147,774	404.86	0.27	116.94
Central Region	1.51	1.10	0.28	16.68	12.14	3.06	1,620	1.45	159.90	131,895	361.36	0.22	110.52
Western Region	2.07	1.48	0.43	3.30	2.36	0.69	774	1.76	28.05	70,770	193.89	0.25	15.95
Economic level													
High	1.50	0.87	0.36	6.51	3.78	1.55	1,640	1.36	59.02	198,092	542.72	0.33	43.35
Middle	2.19	1.72	0.27	16.97	13.35	2.06	1759	1.87	144.93	118,442	324.50	0.18	77.64
Low	1.10	0.84	0.37	6.66	5.12	2.22	477	1.34	81.40	33,905	92.89	0.19	60.62
Total	1.70	1.19	0.32	9.30	6.49	1.77	3,876	1.55	84.66	350,439	960.11	0.25	54.65

**Figure 3:**
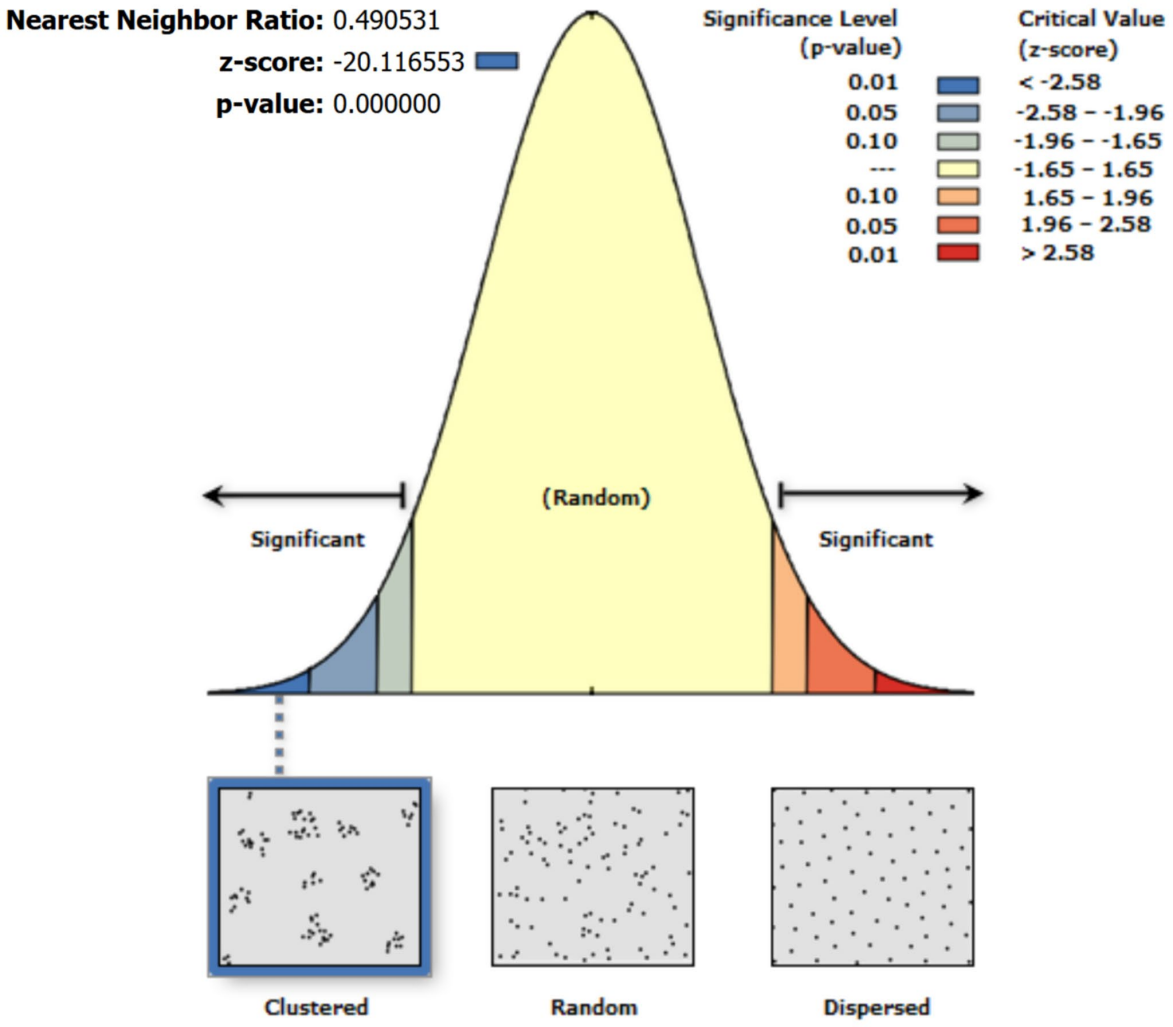
Nearest neighbor analysis of rabies PEP vaccination outpatients in Gansu Province.

**Figure 4:**
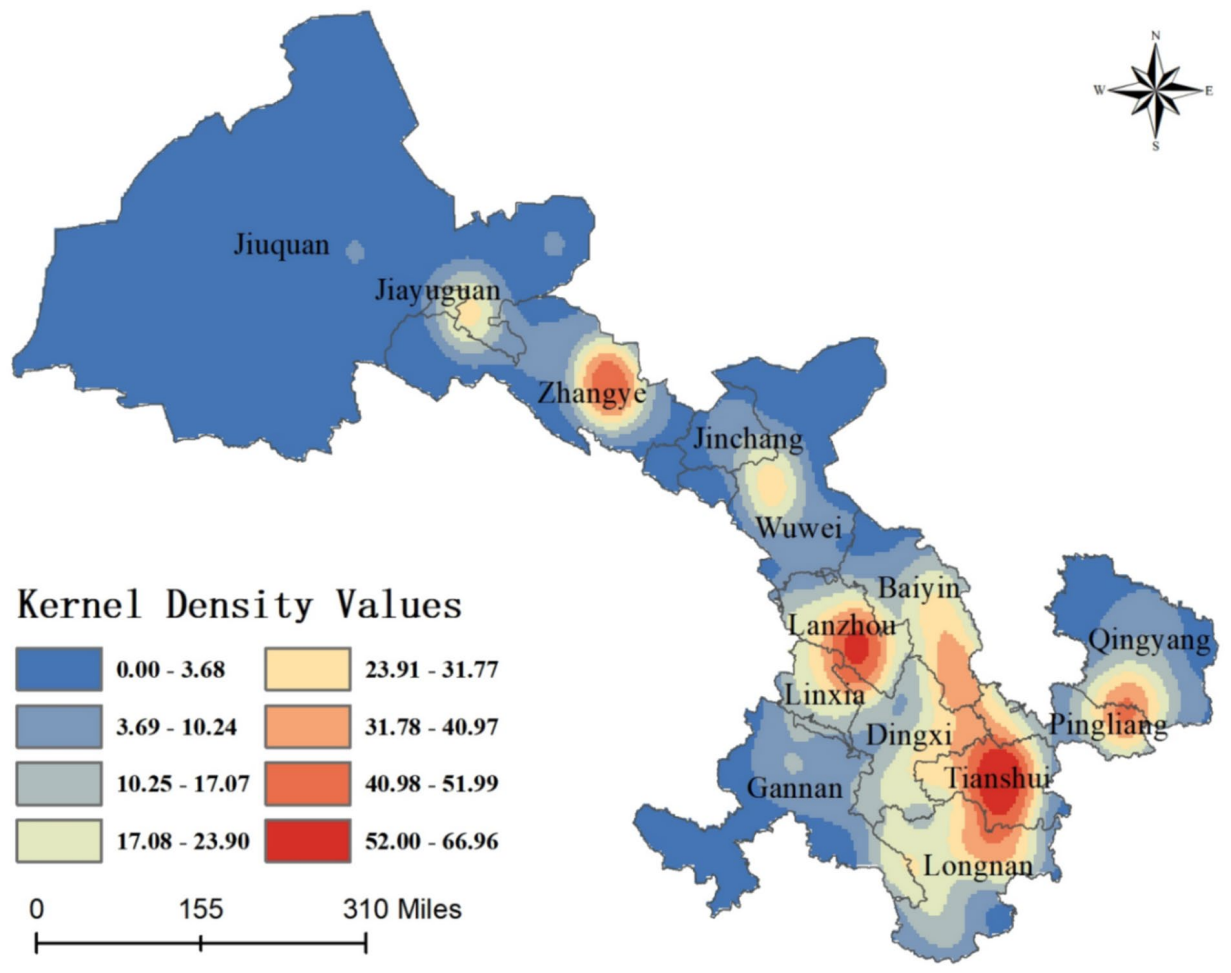
Nuclear density analysis of rabies PEP vaccination clinic in Gansu Province.

To further clarify the spatial clustering characteristics of PEP clinics, kernel density analysis was performed, and the results were consistent with the regional clinic density data in Table 4. The primary hotspot areas with high clinic concentration were concentrated in Lanzhou, Tianshui, Zhangye, and Pingliang. Among these, Tianshui had the highest clinic density (41.58/10^4^ km^2^), Zhangye had a relatively high per capita clinic ownership (3.18/10^5^ population), and Lanzhou and Pingliang also maintained high clinic density levels (36.69/10^4^ km^2^ and 25.07/10^4^ km^2^, respectively). Secondary hotspots, characterized by relatively high clinic concentrations, were observed in parts of Baiyin, Jinchang, and Jiuquan. Notably, Baiyin had a high clinic density (21.69/10^4^ km^2^) and a large annual rabies vaccine administration volume (16,509 doses), which further verified the rationality of its status as a secondary hotspot. The detailed results of the spatial analysis are illustrated in Figures 3, 4.

### 3.3 Traffic costs for residents in each prefecture and City

**Figure 5:**
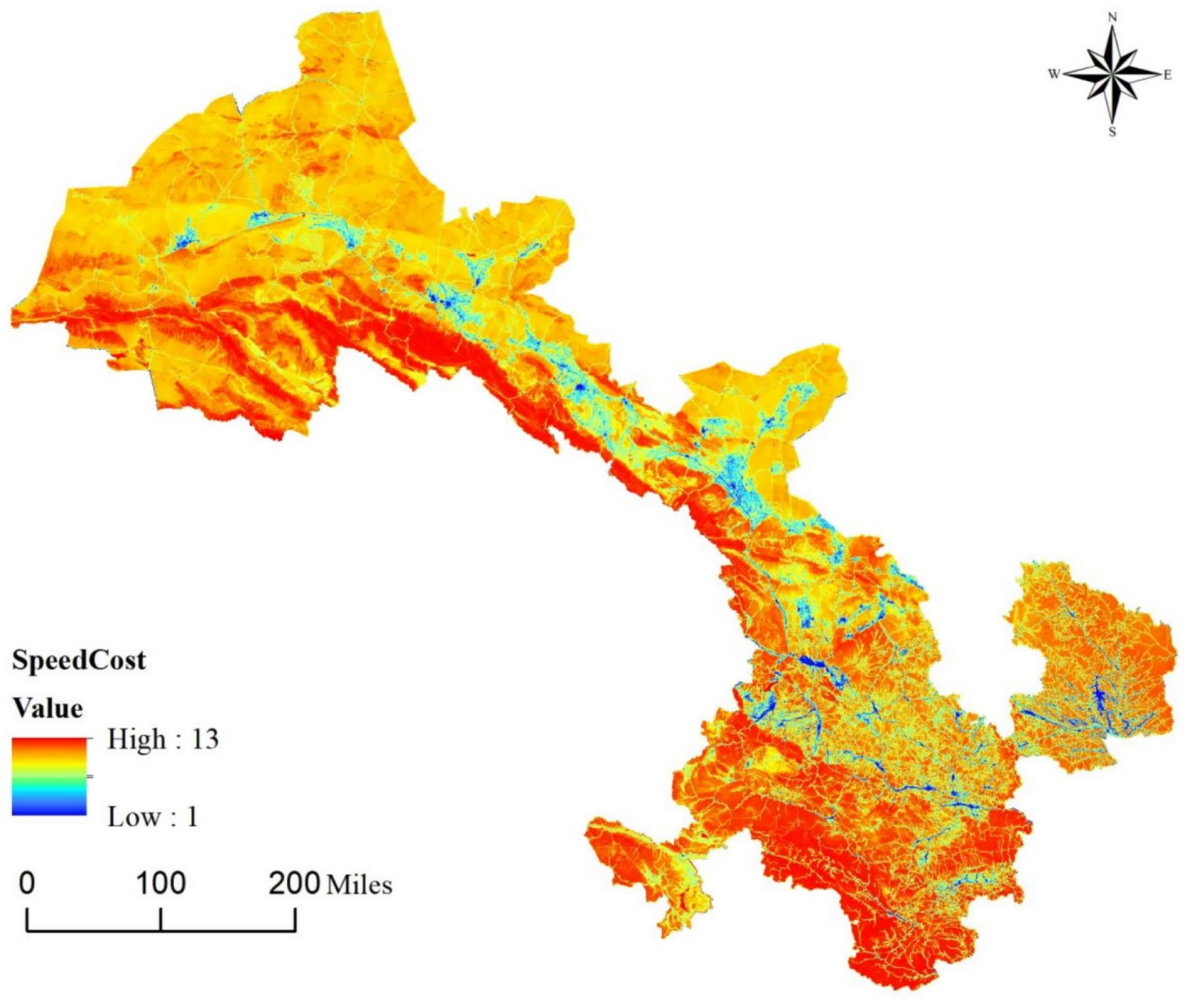
Traffic-cost score in Gansu Province.

**Figure 6:**
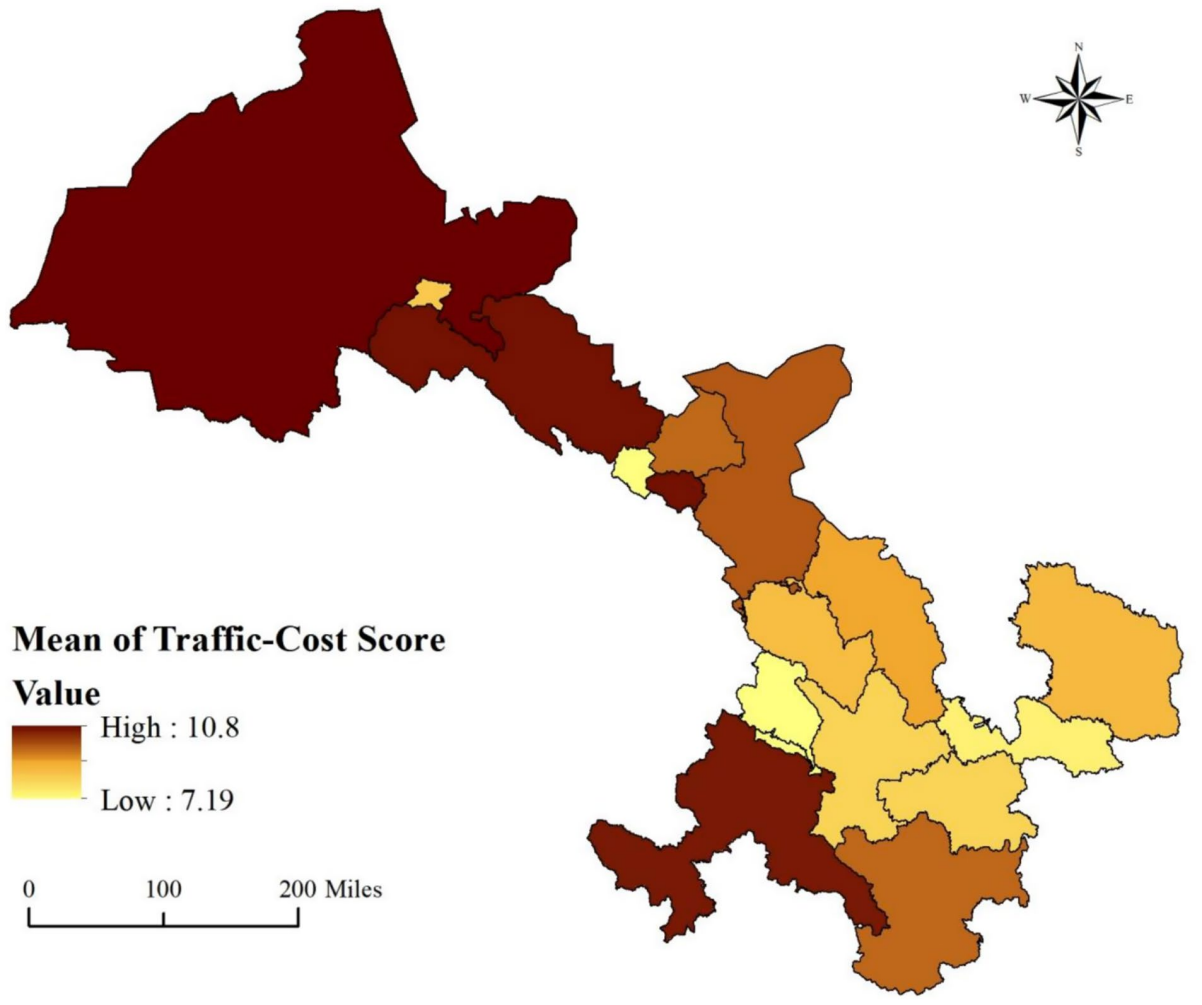
Mean of traffic-cost score in Gansu Province.

As illustrated in Figure 5, the transportation cost layer of Gansu Province characterizes the overall regional transportation conditions. Each grid value represents the transportation cost score of its corresponding area, with lower scores indicating superior transportation accessibility. As shown in Figure 5, the urban oasis areas in the Hexi Corridor region of Gansu Province exhibit low transportation costs, as do the towns and road-connected zones in the Longdong Loess Plateau. In southern Gansu, the Longnan, Tianshui, and Qilian Mountains areas are confronted with elevated transportation costs due to complex terrain, which is attributed to geographical constraints and inadequate transportation accessibility. By contrast, the relatively flat topography of the desert areas in the Hexi Corridor facilitates moderate transportation costs. The top three prefecture-level administrative regions with the best transportation accessibility are Linxia (7.19 ± 4.53), Pingliang (7.56 ± 4.28), and Tianshui (8.10 ± 4.39). Conversely, the three regions with the poorest transportation accessibility are Jiuquan (10.80 ± 2.10), Zhangye (10.68 ± 2.87), and Gannan (10.60 ± 3.13) (Figure 6).

**Figure 7:**
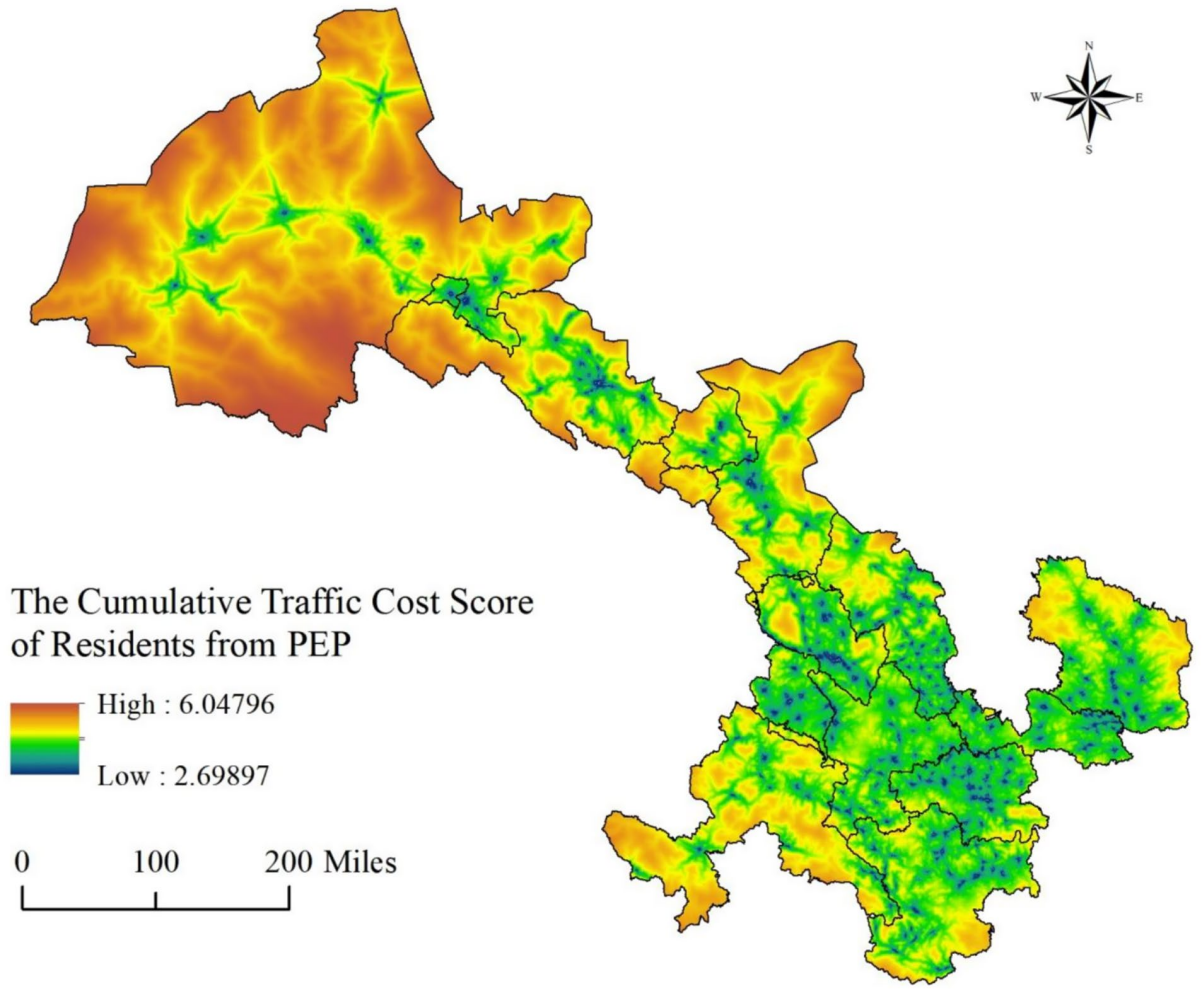
The cumulative traffic cost score of residents from PEP.

The cumulative transportation cost scores for residents to reach their nearest rabies PEP clinics are presented in Figure 7. These scores were ranked in ascending order and visualized with a color gradient shifting from dark blue to green, yellow, and reddish-brown, where lower cumulative transportation cost scores correspond to better accessibility to PEP clinics. Dark blue dots on the map denote the geographic locations of rabies PEP clinics. As shown in Figure 7, green areas extend outward along road networks from the dark blue zones (i.e., clinic locations), and this spatial distribution pattern is particularly prominent in the Hexi Corridor and Gannan. In contrast, the central and eastern Loess Plateau features a higher population density, a more comprehensive road network, and a substantially greater number of PEP clinics than the Hexi Corridor. Accordingly, the green zones in this region exhibit a patchy distribution that is highly consistent with the spatial layout of the transportation network. Among all prefectures, Jiuquan (5.30 ± 0.35), Zhangye (4.98 ± 0.41), and Wuwei (4.93 ± 0.38) had the highest mean cumulative transportation cost scores, while Pingliang (4.40 ± 0.30), Tianshui (4.38 ± 0.36), and Linxia (4.38 ± 0.31) recorded the lowest scores (Table 5).

**Table 5:** Transport cost to the nearest rabies PEP clinic.

Prefecture	Comprehensive transport cost score		Cumulative transport cost score to the nearest rabies PEP clinic	
	Mean	SD	Geometric mean	Geometric SD
Jiuquan	10.80	2.1	5.30	0.35
Zhangye	10.68	2.87	4.98	0.41
Gannan	10.60	3.13	4.91	0.32
Wuwei	9.81	3.12	4.93	0.38
Longnan	9.67	4.31	4.61	0.35
Jinchang	9.66	3.19	4.78	0.37
Baiyin	9.01	3.65	4.55	0.38
Qingyang	8.63	3.81	4.67	0.35
Lanzhou	8.52	3.88	4.51	0.40
Jiayuguan	8.33	4.17	4.52	0.36
Dingxi	8.11	4.17	4.50	0.29
Tianshui	8.10	4.39	4.38	0.36
Pingliang	7.56	4.28	4.40	0.30
Linxia	7.19	4.53	4.38	0.31

## 4 Discussion

**Figure 8:**
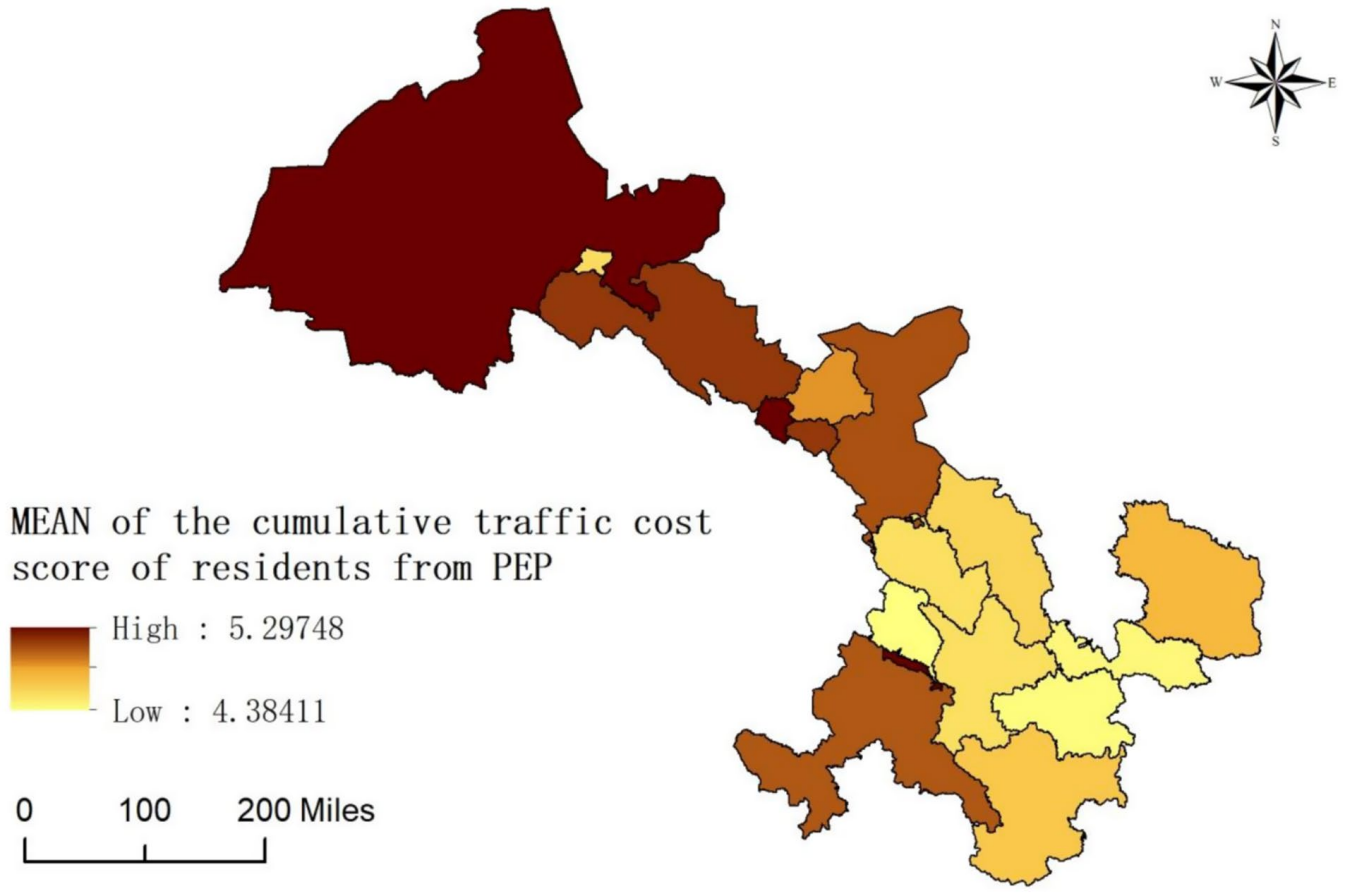
Mean of the cumulative traffic cost score of residents from PEP.

Rabies remains a persistent threat to public health. As one of the countries with high rabies incidence, China has achieved remarkable progress through comprehensive prevention and control measures, with reported cases continuously declining for over a decade (36), Gansu Province, rabies cases were documented in certain counties of Longnan and Zhangye during the 1980s (37). Currently, Gansu Province is characterized by low and sporadic rabies incidence, mainly due to complex terrain, uneven dog vaccination, irregular post-exposure disposal, and occasional cross-regional virus introduction. However, According to Gansu’s surveillance data, the annual exposure rate was 388.72 per 100,000 population from 2019 to 2025. Consequently, it becomes particularly crucial for Gansu Province to further enhance standardized post-exposure management for rabies prevention (Figure 8).

The findings of this study demonstrate that the distribution of rabies post-exposure prophylaxis (PEP) clinic resources in Gansu Province exhibits multidimensional disparities. Western regions and medium-economic-level areas exhibited relatively higher numbers in terms of clinic availability per capita (2.07 and 2.19 clinics per 100,000 population, respectively). Geo spatial analysis revealed a clustered distribution pattern in eastern regions (20.61 clinics per 10,000 km^2^) and sparse distribution in western regions (3.30 clinics per 10,000 km^2^). Notably, medium-economic-level areas have a higher clinic density compared to high-economic-level regions. Regarding human resource allocation, eastern regions maintained a relatively higher density of vaccination personnel per unit area. While western regions showed a reasonable per capita allocation, their personnel distribution by geographic area was markedly sparse. Medium-economic-level areas displayed more balanced human resource allocation.

Significant variations were observed in service provision across different regions and economic levels. In most areas, the proportion of rabies PEP clinics offering wound management services exceeded those providing passive immunizing agents. This discrepancy may be attributed to the prohibitive costs of passive immunizing agents, resulting in limited availability at township health centers and community health service facilities (38). This suggests distinct disparities in the service priorities of rabies PEP clinics across regions with varying economic development levels, which may be associated with population distribution and healthcare resource allocation. In low-economic-level regions of Gansu Province, the total number of PEP clinics is relatively small (39 clinics). Among these, 76.92% are capable of providing wound management services, while only 33.33% can offer passive immunizing agents. It is evident that in regions with lower economic development, due to the limited number of PEP clinics, there is a greater need for clinics that provide comprehensive services. Notably, Gannan and Jinchang, as low-economic-level areas, have relatively high transportation cost scores. Although the proportion of clinics offering passive immunization is relatively high in these areas, their small total number has, to a certain extent, increased transportation costs.

This study employed both density and kernel density metrics to analyze the geographical distribution of rabies PEP clinics. The findings indicate that both density and kernel density values for clinics with varying service capabilities were consistent across different prefectures and cities. Eastern regions exhibited higher clinic density and kernel density, reflecting a more concentrated distribution of rabies PEP facilities. In contrast, western regions showed lower density and kernel density values, indicating a sparser distribution of rabies PEP clinics. Medium-economic-level areas were predominantly located in the eastern regions, which aligns with the geographic disparities in healthcare resource allocation across different regions. These results are consistent with the findings of Wang Jingwen et al., who reported similar variations in the quantity and density of health resources across different regions of Gansu Province (39). The results demonstrate that both density and kernel density metrics exhibit consistency in evaluating the geographical distribution of healthcare resources. However, kernel density calculation is influenced by the distance and quantity of nearby clinics, reflecting the uneven distribution of clinic density within the same administrative region. This spatial characteristic enables residents to access rabies PEP services across administrative boundaries. For instance, while in remote rural areas of Baiyin and Dingxi, where regional density of PEP clinics is low, the kernel density is also low,and maintain limited capacity for wound management and passive immunization agent provision, residents can utilize these services from neighboring clinics in Lanzhou City. Lanzhou, with its stronger PEP service capacity, may serve as a regional hub, extending support to Baiyin and Dingxi to ensure timely and standardized post-exposure care. Such measures help address inappropriate wound management, a key driver of sporadic rabies cases, and accommodate Gansu’s complex terrain and dispersed population patterns, ultimately reducing rabies transmission risks and strengthening public health protection. Therefore, when there are no geographical restrictions on healthcare resource utilization, kernel density can serve as a substitute for density to measure the geographic distribution of healthcare resources.

Gansu Province is characterized by complex and diverse topography, with a complete range of landforms distributed in an interlaced pattern. In this study, we integrated multiple geographical factors, including terrain elevation, landform characteristics, transportation networks, and population density, to calculate travel cost metrics for PEP clinic accessibility. The results demonstrate regional disparities: the eastern region had the lowest transportation costs, followed by the central region, while the western region had the highest. According to the 2023 GDP rankings released by the Gansu Provincial Bureau of Statistics, the economic performance followed the order central > eastern > western, with the western region having the lowest GDP and highest transportation costs. This finding is consistent with Wang’s study (40), which confirmed through modeling that the spatial accessibility of healthcare resources is positively correlated with local GDP. A comparison of rabies PEP clinic density and cumulative transportation costs across the province revealed a positive correlation between rabies PEP clinic density and transport accessibility in all cities and prefectures except Jiayuguan. Although Jiayuguan had a low clinic density, its high transport accessibility was attributed to low transportation costs. This finding aligns with Pancirera’s study (13), which demonstrated that improved transportation significantly enhanced healthcare facility accessibility in Bangladesh without a notable increase in the number of facilities.

This survey through analysis of the basic status of rabies exposure clinics in Gansu Province, combined with kernel density and transportation cost-distance analyses, reveals shortcomings in the standardized construction of rabies PEP clinics in the province. It is recommended to rationally plan clinic layouts, strengthen the service capacity building of clinics in economically underdeveloped and transportation-inconvenient areas, and enhance overall service capabilities. In rabies-endemic areas, when it is not feasible to improve the transportation network, rabies prevention and control can be enhanced through measures such as strengthening animal management, popularizing health education, and optimizing vaccination services.

## 5 Conclusion

Through analysis of the basic status of rabies exposure clinics in Gansu Province, combined with kernel density and transportation cost-distance analyses, reveals shortcomings in the standardized construction of rabies PEP clinics in the province. Strategic recommendations are proposed for rabies-endemic areas: It is recommended to rationally plan clinic layouts, strengthen the service capacity building of clinics in economically underdeveloped and transportation-inconvenient areas, and enhance overall service capabilities. When unable to improve transportation networks, rabies prevention and control can be enhanced through measures such as strengthening animal management, popularizing health education, and optimizing vaccination services.

## Data Availability

The original contributions presented in the study are included in the article/supplementary material, further inquiries can be directed to the corresponding authors.

## Footnotes

1
https://www.worldpop.org/

2
https://www.esa.int/
